# Comparative Evaluation of Tissue and Systemic Responses to Electrospun Biodegradable Polymer and Polypropylene Implants Using hs-CRP and Immunohistochemical Markers

**DOI:** 10.3390/ijms27073231

**Published:** 2026-04-02

**Authors:** Igor A. Eisenach, Elena L. Lushnikova, Galina A. Lapii, Victor S. Ovchinnikov, Anastasia O. Solovieva, Alexey V. Kuznetsov, Vasiliy A. Naprimerov, Vasiliy A. Kuznetsov

**Affiliations:** 1 Institute of Molecular Pathology and Pathomorphology, Federal Research Center for Fundamental and Translational Medicine, 2 Timakova St., Novosibirsk 630117, Russia; 2 Research Institute of Clinical and Experimental Lymphology, Siberian Branch of Russian Academy of Sciences, 6 Arbuzova St., Novosibirsk 630060, Russia; 3 Federal State Budgetary Educational Institution of Higher Education, Novosibirsk State Medical University, Ministry of Health of Russia, 52 Krasniy Prospekt, Novosibirsk 630091, Russia; 4 The Federal Research Center, Institute of Cytology and Genetics Siberian Branch of Russian Academy of Sciences, 10 Prospekt Lavrentyeva, Novosibirsk 630090, Russia; 5 Ya. Postovsky Institute of Organic Synthesis, Ural Branch of Russian Academy of Sciences, 22/20 Akademicheskaya/S. Kovalevskoi St., Ekaterinburg 620990, Russia

**Keywords:** biodegradable polymer, polypropylene, implant, systemic and tissue responses, inflammation, bioinertness, highly sensitive C-reactive protein (hs-CRP), interleukin-6 (IL-6), CD34

## Abstract

The use of biodegradable polymers for the surgical reinforcement of musculofascial structures is of considerable practical interest. Research into the applicability of biopolymers should be conducted in comparison with polypropylene, a material used in surgery. Using highly sensitive C-reactive protein (hs-CRP) in blood and interleukin-6 (IL-6) and CD34 (an endothelial marker with angiogenic properties) in tissues, we analyzed the systemic and local tissue responses to polypropylene and biodegradable polymer implantation in 42 laboratory rats over a three-month period. The study confirmed good biocompatibility of both polymers. However, the systemic and tissue responses to the biopolymer, as measured by the studied markers, were significantly less pronounced compared to polypropylene. Persistently elevated levels of hs-CRP and CD34-positive cells in the biopolymer group at three months post-implantation were attributed to ongoing biotransformation processes. The mild inflammatory response following biopolymer implantation confirms not only its bioinert properties but also its potential for practical surgical applications. Elevated levels of hs-CRP and CD34-, as well as IL-6-positive cells in the polypropylene group, warrant further investigation of long-term responses to polypropylene as potential contributors to post-implantation complications. The hs-CRP level correlated with tissue markers IL-6 and CD34, suggesting its utility as a criterion for assessing postoperative adaptation to implanted synthetic materials.

## 1. Introduction

Contemporary surgical practice extensively employs various synthetic polymers as prosthetic materials and sutures [1,2]. Investigation of tissue and systemic responses to implanted materials, along with the development of novel surgical materials based on these responses, remains critically important [3,4]. Surgery, as an inherent tissue-altering procedure, initiates inflammatory processes that are intensified and prolonged by implant placement [5,6]. Consequently, tissue bioinertness represents a fundamental requirement for functional implant suitability. Material biocompatibility is assessed by evaluating tissue inflammation using various markers [7,8].

Among these markers, interleukin-6 (IL-6), a pleiotropic cytokine, is one of the most sensitive and readily detectable in both tissues and plasma [9,10]. IL-6 is synthesized by multiple cell types and increases in response to trauma, bacterial infection, and hypoxia, making it potentially useful for postoperative monitoring [11,12]. Any surgical procedure or foreign body implantation induces formation of a restrictive connective tissue capsule accompanied by neoangiogenesis [13,14]. These reparative processes can be effectively monitored using CD34, an endothelial marker with angiogenic properties [15,16]. While tissue markers of inflammation are the most sensitive, routine examination of peri-implant tissues is not feasible in clinical surgery [17,18]. Therefore, blood-based inflammatory markers hold the greatest practical value. To determine correlations between tissue and systemic responses, animal experiments that permit collection of histological material and blood samples are essential [19,20]. High-sensitivity C-reactive protein (hs-CRP) is a highly sensitive, versatile, and widely utilized marker [21,22,23,24]. This protein responds not only to mild bacterial infections but also to minor tissue trauma [25,26,27,28].

Long-term studies have confirmed persistent inflammation around various implants, including silicone, at both tissue and systemic levels [29,30]. This phenomenon is exemplified by the recently recognized adjuvant-induced autoimmune/inflammatory syndrome (ASIA), in which surgically implanted synthetic materials function as adjuvants [31,32]. In this context, biodegradable polymer implants, which undergo complete biotransformation (“dissolution”) and connective tissue replacement, are of significant scientific and practical interest [33,34]. When using a biopolymer, inflammation following implantation culminates in connective tissue formation [35]. Biotransformation is a complex process encompassing not only temporal degradation of polymer chains but also phagocytic absorption of dissociated molecules by various cells [13,36]. Within this context, analysis of inflammatory and neoangiogenesis markers in tissues, as well as systemic responses to biopolymer implantation, warrants investigation [14,37]. For practical surgery, it is crucial to identify systemic markers of inflammation associated with the implantation of synthetic materials, especially new, experimental ones, in correlation with tissue markers. This will allow for the diagnosis of various complications without the need for a biopsy of peri-implant tissue. This formed the goal of our study. Such investigations are most informative when conducted in comparison with widely used surgical materials such as polypropylene, which benefits from extensive practical and scientific experience [38,39]. The objective of this research is to conduct a comparative analysis using IL-6, CD34, and hs-CRP of the tissue and systemic responses to implantation of a biodegradable polymer and polypropylene in an experimental setting.

## 2. Results

### 2.1. Experiment: Systemic Response Level of hs-CRP in Blood (ELISA)

**Figure 1:**
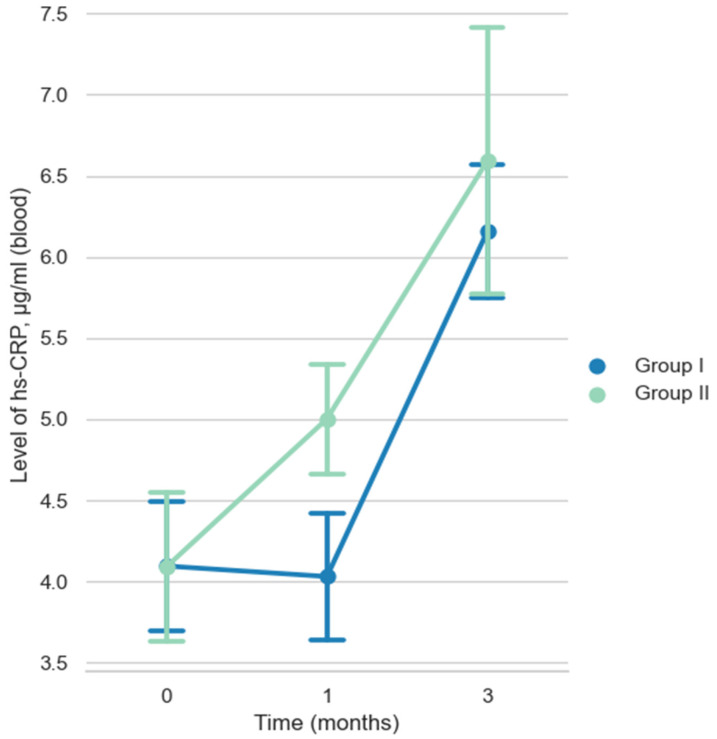
Dynamics of hs-CRP in blood at different times (μg/mL).

No complications were observed in either group of animals with polypropylene **(Group II)** and biopolymer matrix consisting of 65% PCL and 35% PTMC **(Group I)** throughout the experimental period. The mean baseline hs-CRP level in blood before surgery was 4.09 ± 1.95 μg/mL in both groups, as can be seen in Figure 1.

It was slightly lower than the level for this type of intact rats described in the literature [40,41]. One month post-implantation, the mean hs-CRP value in Group I decreased slightly to 4.03 ± 1.79 μg/mL and did not differ significantly from the value at the initial timepoint (before the operation), U = 433 (*p* > 0.05). In Group II, the hs-CRP value one month post-implantation was 5.00 ± 1.55 μg/mL, with a significant difference from the value at the initial timepoint, U = 282 (*p* < 0.05).

The mean hs-CRP value in Group I three months post-implantation increased to 6.16 ± 1.88 μg/mL and significantly differed from the levels at the initial timepoint and at one month post-implantation, U = 160 (*p* < 0.05) and U = 85 (*p* < 0.05), respectively. In Group II, the hs-CRP value three months post-implantation was 6.59 ± 3.75 μg/mL, with a significant difference from the value at the initial timepoint, U = 218 (*p* < 0.05), and without a significant difference from the level at one month post-implantation, U = 181 (*p* > 0.05).

As can be seen from the Graph 1, the hs-CRP level changed differently over time in groups with different implanted materials. One month post-implantation in Group I, the increase in hs-CRP levels was less than in Group II, without a significant difference, 4.03 ± 1.79 μg/mL and 5.00 ± 1.55 μg/mL, respectively, U = 149 (*p* > 0.05). At three months post-implantation, hs-CRP levels in both groups were similar without significant difference, 6.16 ± 1.88 μg/mL and 6.59 ± 3.75 μg/mL, respectively, U = 219 (*p* > 0.05).

### 2.2. Experiment: Tissue Response (Immunohistochemistry)

Three months post-implantation, no external signs of inflammation around the implanted polypropylene **(Group II)** and biopolymer matrix consisting of 65% PCL and 35% PTMC **(Group I)** were detected in any animal on macroscopic examination.

**Figure 2:**
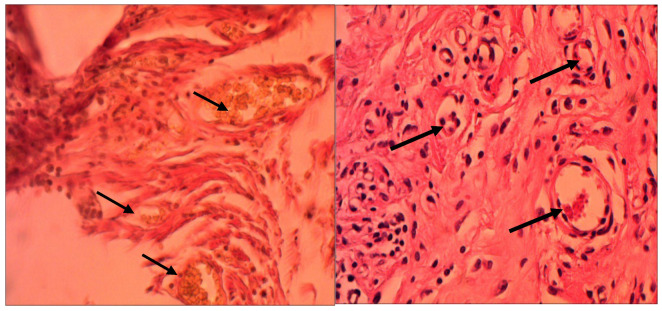
Blood vessels in tissues around polypropylene, left and biopolymer, right (indicated by arrows), three months post-implantation. Van Gieson staining. Magnification 200×.

Connective tissue uniformly encapsulated the implants. An active process of neoangiogenesis with the formation of new vessels also occurred around the polymers in both groups as can be seen in Figure 2.

Immunohistochemical studies revealed varying degrees of elevation of inflammatory markers in peri-implant tissues.

**Figure 3:**
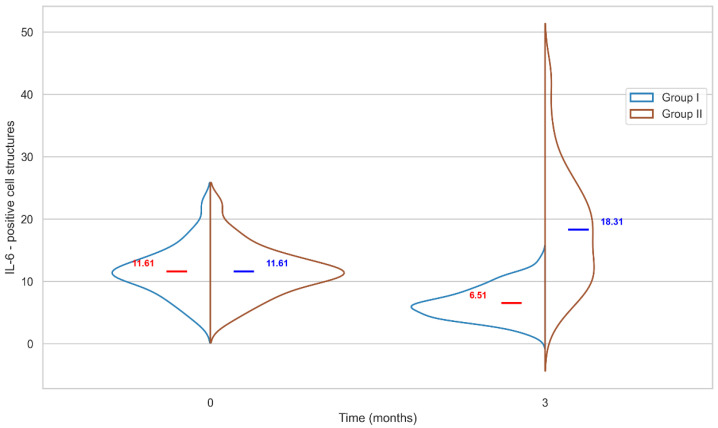
Quantitative changes in IL-6 positive cellular structures in peri-implantation tissues in comparison groups over time.

In Group I, the mean value of IL-6-positive cells at the initial point of the experiment, in intact tissues, was 11.61 ± 4.11, and three months post-implantation was 6.51 ± 2.54, with a significant difference, U = 2838 (*p* < 0.05) as can be seen in Figure 3.

Thus, levels of IL-6-positive cells around the biopolymer significantly decreased post-implantation. In Group II, the mean values of IL-6-positive cells for these timepoints were 11.61 ± 4.11 and 18.31 ± 9.19, respectively, with a significant difference, U = 5444 (*p* < 0.05). The change in the level of IL-6-positive cells in peri-implant tissues for different implants occurred in different directions: around the biopolymer it was decreasing, around polypropylene it was increasing, 6.51 ± 2.54 and 18.31 ± 9.19, with a significant difference, U = 3255 (*p* < 0.05).

**Figure 4:**
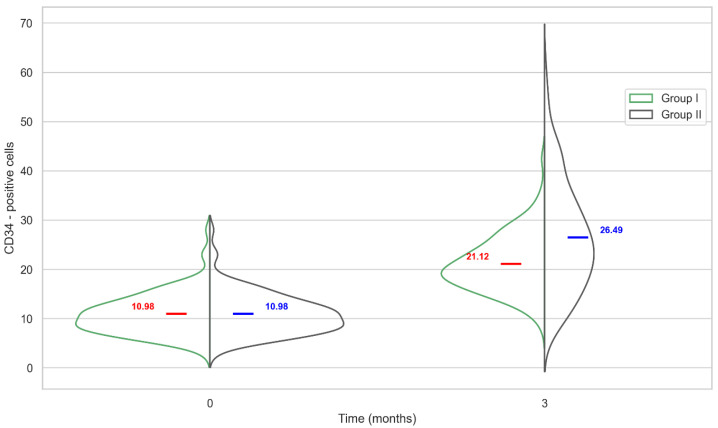
Quantitative changes in CD34-positive cells in peri-implantation tissues in comparison groups over time.

The mean value of CD34-positive cells in Group I for the initial timepoint, in intact tissues, was 10.98 ± 4.21, and three months post-implantation was 21.12 ± 5.76, with a significant difference, U = 1312 (*p* < 0.05) as can be seen in Figure 4.

In Group II, mean values of CD34-positive cells for these timepoints were 10.98 ± 4.21 and 26.49 ± 11.19, respectively, with a significant difference, U = 1601 (*p* < 0.05). The change in the CD34 level in peri-implant tissues for different implants occurred in the same direction: an increase was observed around the biopolymer, and a more significant increase was observed around polypropylene (21.12 ± 5.76 and 26.49 ± 11.19), with a statistically significant difference, U = 14,260 (*p* < 0.05).

As can be seen from the Graph 3, the IL-6 level in the tissues around the biopolymer was significantly lower not only compared to polypropylene but also significantly lower than before the surgery. In tissues around polypropylene, levels of both IL-6- and CD34-positive cells increased, but there were no cases where both markers were at their maximum levels. In Group II specimens 6 and 7, which exhibited maximum levels of CD34-positive cells (40.0 and 40.9, respectively; group mean: 26.5), levels of IL-6-positive cells were below the group mean (12.4 and 17.6, respectively; group mean: 18.3). Conversely, Group II specimens 9 and 18, which exhibited the highest levels of IL-6-positive cells (27.8 and 25.6, respectively; group mean: 18.3), showed levels of CD34-positive cells below the group mean (25.4 and 19.7, respectively; group mean: 26.5).

The hs-CRP levels in both groups were most often higher than the average if there were high values of one of the markers in the peri-implant tissues. For Group I, this was the CD34 marker. For Group II, it could be either marker. For example, specimen 7 in Group II had the highest level of CD34-positive cells in tissue, 40.9, and the highest hs-CRP level in blood, 18.6 μg/mL.

## 3. Discussion

This study confirmed the published data on the good biocompatibility of polypropylene [30,39]. However, experimental results indicate persistent signs of inflammation, such as elevated levels of IL-6- and CD34-positive cells in peri-implant tissues three months post-implantation, when the main stages of inflammation should have concluded [30]. This suggests ongoing inflammation around the implant, dependent on poorly understood factors, potentially contributing to complications consistent with adjuvant-induced autoimmune/inflammatory syndrome (ASIA) [29,31]. This theory is supported by the high level of hs-CRP three months post-implantation in this experiment. These results provide grounds for further study of tissue responses to polypropylene, a material commonly used in surgery, over longer periods. The experimental results suggest that implantation should be conceptualized as an organism-implant system involving long-term adaptive mechanisms, rather than simply as acute inflammation with foreign body encapsulation. This adaptation process may continue throughout the entire period that the implant is present in the body.

The biopolymer used in the experiment was also biocompatible. Tissue responses to this material at three months were less reactive than those to polypropylene. IL-6 levels were significantly lower than both polypropylene levels and baseline intact tissue levels. This may indicate different inflammatory mechanisms and the involvement of other cells in the inflammatory process. These hypotheses are supported by literature data [34,35].

The lower IL-6 level in peri-implantation tissues one month after implantation compared to intact tissues can be explained by two factors. On the one hand, after surgery, as an alteration factor, inflammation and, consequently, markers decrease, and the downward trend in inflammatory mediator levels can drop below physiological values. On the other hand, at this time after implantation, active polymer biotransformation has not yet occurred, and active inflammation is not maintained [1,2,13]. Levels of CD34-positive cells in tissues surrounding the biopolymer increased at three months but were also significantly lower than those of polypropylene. These results support further investigation of biodegradable polymers as implants for various applications in practical surgery.

Despite the small implant size (1 cm^2^) and consequently limited inflammatory field, hs-CRP levels reflected peri-implant tissue processes. The correlation between hs-CRP and inflammation around polypropylene in Group II was linear. Increases in hs-CRP were recorded at one and three months postoperatively, correlating with increased levels of inflammatory markers in peri-implant tissues. Sustained hs-CRP elevation at three months further confirms persistent systemic response to implantation. In the polypropylene group, there were cases of a sharp increase in hs-CRP at three months, potentially reflecting individual hyperreactivity to polypropylene that can lead to various complications. High hs-CRP levels in these cases frequently coincided with elevated levels of IL-6- or CD34-positive cells.

The absence of an increase in hs-CRP during the first month in Group I may be explained by the delayed onset of biopolymer degradation, a phenomenon documented in the literature [33,34]. Consequently, blood hs-CRP levels remained unchanged at one month. By three months, when biotransformation processes were active and tissue levels of CD34-positive cells were elevated, blood hs-CRP levels also increased. The hs-CRP levels increased despite low values of IL-6-positive cells, confirming this protein’s sensitivity to the broader inflammatory milieu. These results support the use of hs-CRP for postoperative monitoring in experimental and clinical settings involving prosthetic materials.

## 4. Materials and Methods

The study included 42 male Wistar rats (3 months old) divided into two groups of 21 animals each. Each animal received a subcutaneous implant (1 × 1 × 0.2 cm) on the dorsum. Group I received a biopolymer matrix consisting of 65% polycaprolactone (PCL) and 35% polytrimethylene carbonate (PTMC). **Electrospun (MECC CO., Ltd., Fukuoka, Japan) using PLC and PTMC polymers (Sigma-Aldrich, St. Louis, MO, USA) prepared the biopolymer by dissolution in chloroform.** Group II received polypropylene mesh having a specific gravity of 28 g/m^2^. The studies were carried out in accordance with the Declaration of Helsinki of the World Medical Association on the humane treatment of laboratory animals, and the directive of the European Community (86/609/EU).

### 4.1. Animal Experiment: Study of hs-CRP (ELISA)

In both groups, blood was collected from the animals’ tails at three timepoints: on the day of surgery before implantation (baseline); one month post-implantation; and three months post-implantation. Blood was collected in test tubes with EDTA, followed by analysis using an ELISA kit for highly sensitive C-reactive protein (hs-CRP) (Elabscience, Wuhan, China). Spectrophotometry was performed using a Multiscan Sky device (Thermo Scientific, Singapore). The hs-CRP measurements obtained at different timepoints and for different groups were compared by mean value to determine significance using the nonparametric Mann–Whitney U test. Baseline hs-CRP values were calculated for all animals from both groups combined. The hs-CRP values at one and three months post-implantation were calculated for each individual group (Groups I and II). Statistical analysis of the material and graphical representation were performed using Statistica 6.4, Matplotlib 3.10.8 and Seaborn 0.13.2, which are specific, recent versions of Python 3.10 data visualization libraries application software package.

### 4.2. Animal Experiment: Tissue Research (Immunohistochemistry)

A small skin sample was obtained from all animals during initial surgery for immunohistological baseline analysis. All animals were euthanized at three months post-implantation. Histological material was collected in the form of biopolymer and polypropylene with surrounding tissues. After fixing the samples in a 10% solution of neutral formalin and standard processing on a Leica TP1020 histological complex (Nußloch, Germany), they were embedded in paraffin blocks. Sections 3–4 μm thick were prepared using a Leica RM2235 rotary microtome (Germany). Paraffin sections were stained with hematoxylin and eosin in a Leica ST5010 apparatus (Nußloch, Germany) and according to van Gieson. Histological preparations were examined in an Olympus CX31 microscope with a Nikon DS-Fi1 digital video camera (Tokyo, Japan).

The immunohistochemical procedure, adapted for this laboratory, was performed in accordance with guidelines for immunohistochemical studies and antibody manufacturer recommendations [42,43]. Before the study, prepared sections were deparaffinized and tissue antigens were retrieved in a PT Link module (Dako, Glostrup, Denmark) in citrate buffer (pH 9.0) at 95 °C for 60 min. Endogenous peroxidase was blocked with a 3% H_2_O_2_ solution, followed by protein blocking with serum. Next, tissue sections were incubated with antibodies to IL-6ST rabbit polyclonal (Elabscience, Wuhan, China) and CD34 (clone QBEnd 10) mouse monoclonal (Dako, Denmark). All antibodies were diluted and incubated according to manufacturers’ instructions. A peroxidase-labeled polymer detection system was used for immunostaining. The final step was to counterstain cell nuclei with hematoxylin.

The resulting immunohistochemical analysis was assessed semi-quantitatively. When examining antibody-stained preparations, numerical density—the ratio of positively stained cellular structures—was calculated. For CD34, positive nuclear–cytoplasmic staining was observed, while for IL-6ST, positive membranous-cytoplasmic staining was observed. In the implant area, formed granuloma was assessed, and vessels were counted along the section surface at 200× magnification. Volume density of structures positively stained with IL-6ST and CD34 was counted in 10 fields of view at 400× magnification using a morphometric grid with 121 nodes. Volume density was determined using the formula: Vv = x/n, where x is the number of points per specific structural component and n is the total number of points counted [44].

Mean values were compared using the nonparametric Mann–Whitney U test. Tissue parameter values were compared between baseline (intact tissues) and three months post-implantation. Baseline CD34 and IL-6 values were calculated for all animals from both groups combined. Values at three months post-implantation were calculated for each individual group. Statistical analysis of the material and graphical representation were performed using the Statistica 6.4, Matplotlib 3.10.8 and Seaborn 0.13.2 are specific, recent versions of Python data visualization libraries application software package.

## 5. Conclusions

This experiment confirmed the bioinertness of the biodegradable polymer. Based on hs-CRP, CD34, and IL-6 parameters, both tissue and systemic responses were significantly less pronounced compared to polypropylene (*p* < 0.05), supporting the potential application of this biopolymer in surgical practice. The inflammatory response to the biopolymer was qualitatively distinct from that of polypropylene. Markers were significantly lower in peri-implant tissues and blood around the biopolymer, and levels of IL-6-positive cells remained below baseline throughout the experiment.

Despite the low level of IL-6-positive cells in Group I with biopolymer one month after implantation, hs-CRP effectively reflected inflammatory processes in tissues surrounding both biopolymer and polypropylene. The hs-CRP levels were influenced by other tissue inflammatory markers, particularly CD34, confirming high sensitivity for detecting inflammation around surgical implants. Increased levels of tissue markers of inflammation in both groups three months after implantation were reflected in elevated levels of hs-CRP. Therefore, hs-CRP may be valuable in clinical practice for postoperative monitoring. Some specimens in Group II exhibited markedly elevated levels of CD34-positive cells and hs-CRP, confirming individual hyperreactivity to polypropylene that should be considered in surgical practice.

## Data Availability

The data that support the findings of this study are available from the corresponding author upon reasonable request.

## Abbreviations

The following abbreviations are used in this manuscript:

ASIA	Adjuvant-induced autoimmune/inflammatory syndrome
CD34	Endothelial marker with angiogenic properties
ELISA	Enzyme-linked immunosorbent assay
EDTA	Ethylenediaminetetraacetic acid
Hs-CRP	Highly sensitive C-reactive protein
IL-6	Interleukin-6
PCL	Polycaprolactone
PTMC	Polytrimethylene carbonate
